# *Lonicera flos* and *Curcuma longa* L. extracts improve growth performance, antioxidant capacity and immune response in broiler chickens

**DOI:** 10.3389/fvets.2024.1388632

**Published:** 2024-04-12

**Authors:** Dahai Xu, Xiao Wang, Wanyu Shi, Yongzhan Bao

**Affiliations:** ^1^College of Traditional Chinese Veterinary Medicine, Hebei Agricultural University, Baoding, China; ^2^Hebei Provincial Veterinary Biotechnology Innovation Center, Baoding, China; ^3^Hebei Provincial Traditional Chinese Veterinary Medicine Technology Innovation Center, Baoding, China

**Keywords:** antioxidant capacity, broiler, growth, immunity, plant extract

## Abstract

Alternatives to antibiotics are urgently needed to maintain broiler growth and health. The present study was conducted to evaluate the effects of *Lonicera flos* and *Curcuma longa* L. extracts (LCE) as antibiotic substitutes on growth performance, antioxidant capacity and immune response in broilers. A total of 480 one-day-old female broilers (WOD168) were allocated to 3 treatments with 5 replicates of 32 birds for 35 days. The 3 treatments were: an antibiotic-free basal diet (control, CON), CON +50 mg/kg spectinomycin hydrochloride and 25 mg/kg lincomycin hydrochloride (ANT), CON +500 mg/kg LCE (LCE). During the entire experimental period, supplementation of ANT and LCE increased (*p* < 0.01) average daily gain (ADG) and decreased (*p* < 0.05) feed conversion ratio (FCR), thereby resulting in greater final body weight (BW) compared with CON. Dietary LCE supplementation increased (*p* < 0.05) serum (glutathione peroxidase) GSH-Px, (superoxide dismutase) SOD and total antioxidant capacity (T-AOC) activities, and decreased (*p* < 0.05) serum malonaldehyde (MDA) concentration at day 35 compared with CON. There was no significant difference in serum catalase (CAT) activity among treatments. Birds in LCE group had lower (*p* < 0.05) MDA concentration and higher SOD activity in liver than those in CON and ANT groups at day 35. Birds in LCE group had higher (*p* < 0.05) phagocytic index and serum antibody titers to Newcastle disease virus (NDV) than those in CON group. Lower (*p* < 0.05) concentrations of pro-inflammatory cytokines and higher (*p* < 0.05) concentrations of anti-inflammatory cytokines in serum and liver were observed in birds fed LCE diet than those fed CON diet. In conclusion, dietary supplementation of LCE improved growth performance by enhancing antioxidant capacity, strengthening immune system and alleviating inflammation, which has potential as antibiotic alternatives.

## Introduction

Antibiotics have been widely used in poultry industry due to their well-known growth promoting properties (1, 2). Over the past decades, the application of in-feed antibiotics has brought dramatic improvements in poultry productivity by ameliorating growth performance, modifying the intestinal microbiota, preventing bacterial infections and reducing mortality (3, 4). Unfortunately, long-term overuse of in-feed antibiotics has caused bacterial resistance and drug residue, which are hazardous to human and animal health (5, 6). As a consequence, antibiotic usage in poultry feeds has been gradually banned worldwide (2). In China, the use of antibiotics in poultry feeds has been banned since 2020. However, the complete withdrawal of in-feed antibiotics has undoubtedly resulted in compromised growth performance and high mortality (7, 8). Hence, it is vitally important to develop alternatives for antibiotics to improve growth and health of broilers.

Phytogenic compounds are natural bioactive compounds derived from plants that have beneficial effects on the health and growth of animals (9). Recently, phytogenic compounds have attracted widespread attention as substitutes for antibiotics due to their low toxic and residue free properties (10, 11). *Lonicera flos* (“Shanyinhua” in Chinese) is the dried flower buds or flowers of four *Lonicera* plants (*Lonicera macranthoides*, *Lonicera hypoglauca*, *Lonicera confusa*, and *Lonicera fulvotnetosa*) (12), and has been widely used as a heat-clearing and detoxifying medicine for thousands of years in China (13). The main active constituent of *Lonicera flos* is chlorogenic acid (CGA), which has been proved to possess antioxidant, anti-inflammatory and anticancer properties (14–16). *Curcuma longa* L., commonly known as turmeric, is a rhizomatous herb belonging to the family Zingiberaceae with important medicinal value (17). Curcumin, the primary active constituent of turmeric, is now considered as being responsible for most of the therapeutic effects of turmeric due to its antioxidant, anti-microbial, anti-inflammatory, antiangiogenic and antimutagenic properties (18, 19). In broilers, previous studies have demonstrated that dietary supplementation of CGA or curcumin alleviated stress-induced growth repression and intestinal damage by suppressing inflammation response, improving antioxidant capacity, and enhancing intestinal barrier function (14, 20–22). However, the low bioavailability of CGA and curcumin has proved a challenge, requiring high doses to achieve its benefits (23, 24). A recent *in vitro* study has suggested that CGA potentiates the anti-inflammatory activity of curcumin in LPS-stimulated THP-1 cells (25), indicating that the synergistic combination of CGA and curcumin may be potential alternatives to antibiotics. However, little information is available regarding the effects of co-administration of CGA and curcumin in broiler chickens. Therefore, the present study was conducted to evaluate the effects of a combination of *Lonicera flos* and *Curcuma longa* L. extracts (LCE) as antibiotic alternatives on growth performance, antioxidant capacity and immune response in broilers.

## Materials and methods

All experimental procedures and use of animals in the current study were reviewed and approved by the Institutional Animal Care and Use Committee of Hebei Agricultural University (Baoding, China; No. 2022161).

### Source of *Lonicera flos* and *Curcuma Longa* L. extracts

The LCE product was provided by Centre Technology Co., Ltd. (Beijing, China). The LCE consisted of extracts from *Lonicera flos* and *Curcuma longa* L. in certain proportion. The final concentrations of chlorogenic acid and curcumin were 100 mg/kg and 20 g/kg, respectively.

### Experimental design, diets and husbandry

A total of 480 one-day-old healthy female broilers (WOD168) were obtained from Beijing Huadu Yukou Poultry Industry Co., Ltd. (Beijing, China). The birds were weighed on arrival and randomly assigned to 3 treatments with 5 replicates of 32 birds. The control group (CON) was fed an antibiotic-free basal diet. The antibiotic group (ANT) was fed the basal diet supplemented with 50 mg/kg spectinomycin hydrochloride and 25 mg/kg lincomycin hydrochloride (Sichuan Hengtong Animal Pharmacy Co., Ltd., Neijiang, China). The LCE group was fed the basal diet supplemented with 500 mg/kg LCE. The concentrations of chlorogenic acid and curcumin in the LCE diet were 50 μg/kg and 10 mg/kg, respectively. The feeding program was divided into 2 phases: starter (days 1–21) and finisher (days 22–35). The basal diets for each phase were formulated to meet the nutrient requirements recommended by the National Research Council (NRC, 1994) and manufactured in mash form. The composition and nutrient levels of basal diets were shown in Table 1.

**Table 1:** Ingredient composition and nutrient levels of basal diet (%, as-fed basis).

Item	Day 1–21	Day 22–35
Corn	52.50	58.80
Soybean meal	40.00	33.80
Soybean oil	3.00	3.00
Dicalcium phosphate	1.90	1.80
Limestone	1.08	1.22
Salt	0.37	0.37
L-lysine HCl	0.05	0.03
DL-methionine	0.19	0.07
Choline chloride	0.11	0.11
Premix^a^	0.80	0.80
Calculated nutrient composition	0.00	0.00
Metabolic energy (MJ/kg)	12.42	12.62
Calcium	1.00	1.02
Available phosphorus	0.44	0.42
Analyzed nutrient composition	0.00	0.00
Crude protein	22.27	19.24
Lysine	1.34	1.15
Methionine	0.55	0.40

^a^Premix supplied per kg diet: vitamin A, 6,141 IU; vitamin D, 1,782 IU; vitamin E, 8.0 mg; vitamin K_3_, 1.8 mg; thiamine, 0.65 mg; riboflavin, 3.9 mg; pyridoxine, 2.1 mg; cobalamin, 10.0 μg; nicotinic acid, 18.1 mg; pantothenic acid, 6.7 mg; folic acid, 0.6 mg; biotin, 70 μg; choline chloride, 332 mg; iron, 60.9 mg; zinc, 65.8 mg; manganese, 62.3 mg; copper, 6.0 mg; selenium, 0.21 mg; iodine, 0.90 mg.

All birds were housed in two-tier cages (1.00 m × 1.25 m × 0.45 m) in an environmentally controlled room. A light schedule of 23 h light and 1 h dark was used throughout the experimental period. Room temperature were maintained at 33°C for the initial 3 days and then gradually decreased by 3°C every week to reach a final temperature of 24°C. The relative humidity was maintained at 70% within the first 3 days and at 60% afterward. *Ad libitum* water and feed were provided throughout the experimental period. Birds were vaccinated against Newcastle disease (ND), Infectious bronchitis, Avian influenza (H9 subtype; AI) and Infectious bursal disease on day 1.

### Growth performance

At 1, 21 and 35 days of age, body weight (BW) and feed intake were recorded on a cage basis to calculate average daily gain (ADG), average daily feed intake (ADFI) and feed conversion ratio (FCR). Mortality was recorded daily to modify the performance parameters.

### Sample collection

At 21 and 35 days of age, 5 birds (1 birds per replicate cage) from each treatment were randomly selected after 12 h fasting. Blood samples were collected from the wing vein into tubes with or without EDTA to yield whole blood and serum, respectively. Whole blood was immediately transported to the lab for peripheral blood lymphocyte proliferation analysis. Serum was obtained by centrifugation at 3,000 × g for 15 min at 4°C and stored at −80°C until analysis. Subsequently, birds were sacrificed by cervical dislocation. The liver was collected, snap frozen in liquid nitrogen, and stored at −80°C until analysis.

### Antioxidant capacity and inflammatory cytokines

The liver tissue (1 g) was homogenized in 9 mL of ice-cold phosphate buffer saline to prepare the liver tissue homogenate and then centrifuged at 3,000 × g for 15 min at 4°C. The supernatant was collected for further analysis. Total protein concentration in liver tissues was measured by the bicinchoninic acid (BCA) method as described previously (26).

Total-antioxidant capacity (T-AOC, No. A015-2-1), malondialdehyde (MDA, No. A003-1-2) concentration, activities of glutathione peroxidase (GSH-Px, No. A005-1-2), superoxide dismutase (SOD, No. A001-1-2) and catalase (CAT, No. A007-1-1) in serum and liver tissues were determined with commercially available kits (Nanjing Jiancheng Bioengineering Institute, Nanjing, China) according to the manufacturer’s instructions. Results in liver tissues were normalized against the corresponding total protein concentrations and expressed as units/mg protein.

Interleukin-6 (IL-6, No. ml059839), interleukin-10 (IL-10, No. ml059830), tumor necrosis factor-α (TNF-α, No. ml002790), interferon-γ (IFN-γ, No. ml042758) and prostaglandin E2 (PGE-2, No. ml058373) concentrations in serum and liver tissues were analyzed by commercial ELISA kits (Shanghai Enzyme-linked Biotechnology Co., Ltd., China) following the manufacturer’s instructions. Values were normalized to total protein concentrations in liver tissues, and given as units/g protein.

### Peripheral blood lymphocyte isolation and proliferation

Peripheral blood lymphocytes were isolated from whole blood using density gradient centrifugation (Solarbio, Beijing, China). Then collected lymphocytes were washed three times with RPMI 1640 medium and suspended in RPMI 1640 medium (Solarbio, Beijing, China) supplemented with 10% fetal bovine serum, 2 mM L-glutamine, 100 IU/mL penicillin, and 100 μg/mL streptomycin. Cell number and viability were determined using trypan blue staining (Sigma, Germany), and the final concentration of lymphocytes was adjusted to 1 × 10^7^ viable cells/mL.

Lymphocyte proliferation was measured by 3-(4,5-dimethylthiazol-2 yl)-2,5-diphenyl tetrazolium bromide (MTT) assay (Solarbio, Beijing, China). Briefly, 100 μL of lymphocyte suspension and 100 μL of RPMI 1640 medium with or without 90 μg/mL concanavalin A (Con A; Sigma Chemical Co., St. Louis, MO) was added to each microwell in a 96-well plate in triplicate. After incubation for 72 h at 39°C in a 5% CO_2_ incubator, 10 μL MTT was added into each well, incubated for 4 h, and then 100 μL of dimethyl sulfoxide was incorporated into each well and shaken until complete dissolution. Light absorbance serving as an index of lymphocyte proliferation was determined at 570 nm using a microplate reader (Bio-Tek, United States) and expressed as a mean stimulation index.

### Mononuclear phagocytosis assessment

The phagocytic activity of reticuloendothelial system was evaluated by carbon clearance assay as described previously (27). Briefly, the black ink (Pelikan, Hanover, Germany) was centrifuged at 3000 × g for 30 min to collect colloidal carbon. Then 10 birds from each treatment were weighed and injected (0.1 mL/300 g of BW) with colloidal carbon via wing vein at 21 and 35 days of age. Blood samples were collected at the time intervals of 3 and 15 min post injection, and then were immediately suspended into 4 mL solution of 0.1% sodium carbonate solution. The absorbance was measured at 640 nm in a NanoDrop ND-1000 spectrophotometer (NanoDrop products, Wilmington, DE, United States). After then, birds were sacrificed, and spleen and liver were weighed. Phagocytic index was calculated using the following equations (27):


$$\begin{array}{c} \mathrm{Phagocytic}\;\mathrm{index}=\left[\log\;OD3-\log\;OD15/\left(T2-T1\right)\right]\wedge^{1/3}\\ \times \;BW/\left(\mathrm{liver}\;\mathrm{weight}+\mathrm{spleen}\;\mathrm{weight}\right) \end{array}$$

where OD3 is the absorbance at 3 min, OD15 is the absorbance at 15 min, T1 is the first time point of blood collection, and T2 is the last time point of blood collection.

### Serum antibody titers against NDV

At 21 and 35 days of age, serum antibodies against NDV were determined by hemagglutination inhibition (HI) assays. HI tests were carried out by using serial 2-fold dilutions of serum and 4 hemagglutination units of the NDV antigen (Harbin Weike Biotechnology Co. Ltd., China). The geometric mean titer was expressed as reciprocal log2 values of the highest dilution that displayed HI.

### Statistical analysis

Date were subjected to a one-way ANOVA using the GLM procedures of SAS (version 9.2; SAS Inst. Inc., Cary, NC, United States) followed by Tukey’s tests. Each cage was defined as an experimental unit. Treatment was considered a fixed effect and bird was as a random effect. Significant difference was declared at *p* < 0.05.

## Results

### Growth performance

The results of growth performance in broiler chickens are presented in Table 2. There was no significant difference in initial BW among treatments. However, birds in ANT and LCE groups had similar BW, but greater (*p* < 0.01) than those in CON group at day 21 and 35. From day 1 to 21, dietary supplementation of LCE increased (*p* < 0.05) ADG and decreased (*p* < 0.01) FCR, while dietary supplementation of ANT only increased (*p* < 0.01) ADG when compared with CON. No difference was observed in ADFI among treatments. From day 22 to 35, these performance parameters did not differ significantly among treatments. During the entire experimental period, dietary ANT and LCE supplementation resulted in a significant increase (*p* < 0.01) in ADG and a significant decrease (*p* < 0.05) in FCR compared with CON.

**Table 2:** Effects of dietary LCE supplementation on growth performance in broiler chickens.

Items	CON	ANT	LCE	SEM	*p*-value
*BW (g)*					
Day 1	39.88	40.12	39.93	1.04	0.99
Day 21	365.52^b^	391.35^a^	408.77^a^	6.46	<0.01
Day 35	797.90^b^	832.15^a^	862.22^a^	8.51	<0.01
*ADG (g/day)*					
Day 1–21	15.34^b^	16.67^a^	17.55^a^	0.35	<0.01
Day 22–35	30.96	32.11	32.71	0.56	0.12
Day 1–35	21.07^b^	22.14^a^	22.90^a^	0.26	<0.01
*ADFI (g/day)*					
Day 1–21	26.36	26.33	26.18	0.21	0.82
Day 22–35	55.79	56.09	58.38	0.78	0.07
Day 1–35	37.13	36.88	37.51	0.27	0.28
*FCR*					
Day 1–21	1.73^a^	1.58^ab^	1.49^b^	0.04	<0.01
Day 22–35	1.81	1.75	1.79	0.04	0.58
Day 1–35	1.77^a^	1.67^b^	1.64^b^	0.03	0.01

^a,b^Means in the same row without a common superscript differ significantly (*p* < 0.05). CON, basal diet; ANT, basal diet supplemented with 50 mg/kg spectinomycin hydrochloride and 25 mg/kg lincomycin hydrochloride; LCE, basal diet supplemented with 500 mg/kg LCE.

### Serum antioxidant capacity

The effects of LCE on serum antioxidant capacity in broiler chickens are shown in Table 3. At day 21, serum MDA concentration, CAT activity and T-AOC were not influenced by dietary treatments. However, birds in LCE group had higher (*p* < 0.05) serum activities of GSH-Px and SOD than those in CON group, but similar with those in ANT group. At day 35, dietary LCE supplementation decreased (*p* < 0.05) serum MDA concentration, and increased (*p* < 0.05) serum SOD activity and T-AOC compared to other groups. Besides, supplementation of ANT and LCE increased (*p* < 0.05) serum GSH-Px activity when compared with CON. Serum CAT activity was not influenced by dietary treatments.

**Table 3:** Effects of dietary LCE supplementation on serum antioxidant capacity in broiler chickens.

Items	CON	ANT	LCE	SEM	*p*-value
*Day 21*					
MDA (nmol/mL)	1.89	1.91	1.64	0.19	0.57
GSH-Px (μmol/L)	1146.28^b^	1282.09^ab^	1353.04^a^	44.21	0.01
CAT (U/mL)	1.32	1.31	1.40	0.10	0.78
SOD (U/mL)	845.40^b^	856.81^ab^	885.09^a^	11.23	0.03
T-AOC (mmol/L)	0.58	0.59	0.59	0.01	0.11
*Day 35*					
MDA (nmol/mL)	1.79^a^	1.78^a^	1.35^b^	0.10	0.01
GSH-Px (μmol/L)	1579.83^b^	1712.00^a^	1814.96^a^	30.46	<0.01
CAT (U/mL)	3.90	3.76	4.25	0.14	0.06
SOD (U/mL)	868.22^b^	870.20^b^	915.84^a^	7.36	<0.01
T-AOC (mmol/L)	0.73^b^	0.73^b^	0.78^a^	0.01	<0.01

^a,b^Means in the same row without a common superscript differ significantly (*p* < 0.05). CON, basal diet; ANT, basal diet supplemented with 50 mg/kg spectinomycin hydrochloride and 25 mg/kg lincomycin hydrochloride; LCE, basal diet supplemented with 500 mg/kg LCE.

### Peripheral blood lymphocyte proliferation

**Figure 1:**
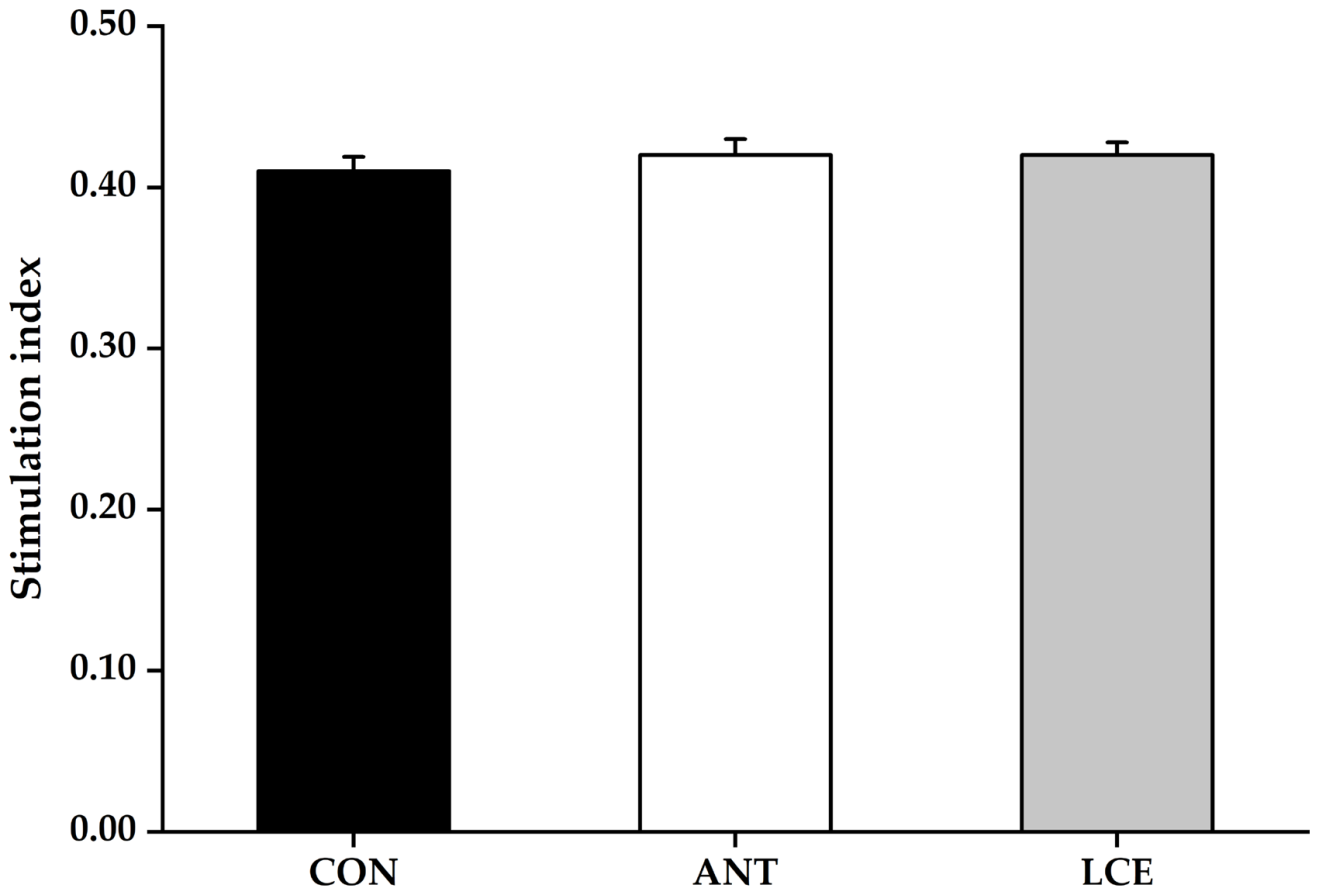
Effects of dietary LCE supplementation on peripheral blood lymphocyte proliferation in broiler chickens at day 35. CON, basal diet; ANT, basal diet supplemented with 50 mg/kg spectinomycin hydrochloride and 25 mg/kg lincomycin hydrochloride; LCE, basal diet supplemented with 500 mg/kg LCE. Values are expressed as mean ± SEM, *n* = 5.

Effects of dietary LCE supplementation on peripheral blood lymphocyte proliferation in broiler chickens at day 35 are presented in Figure 1. There was no significant difference in stimulation index of peripheral blood lymphocytes among treatments at day 35.

### Phagocytic index

**Figure 2:**
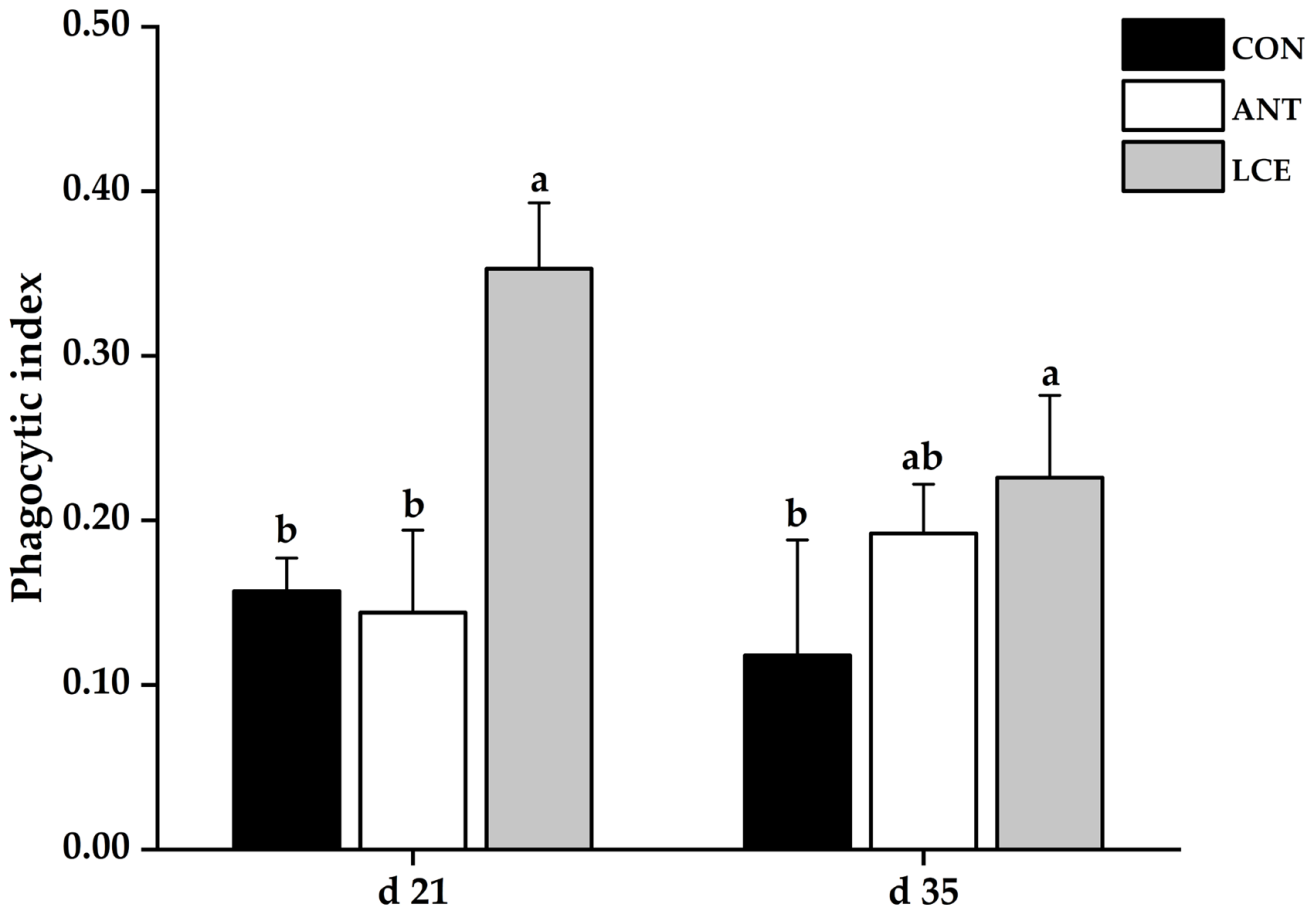
Effects of dietary LCE supplementation on phagocytic index in broiler chickens. CON, basal diet; ANT, basal diet supplemented with 50 mg/kg spectinomycin hydrochloride and 25 mg/kg lincomycin hydrochloride; LCE, basal diet supplemented with 500 mg/kg LCE. Values are expressed as mean ± SEM, *n* = 5. ^a,b^Means without a common superscript differ significantly (*p* < 0.05).

Effects of dietary LCE supplementation on phagocytic index in broiler chickens are presented in Figure 2. At day 21, dietary LCE supplementation significantly increased (*p* < 0.05) phagocytic index when compared with the other groups. At day 35, birds fed LCE diet had higher (*p* < 0.05) phagocytic index than those fed CON, but not different from those fed ANT diet.

### Serum antibody titers to NDV

**Figure 3:**
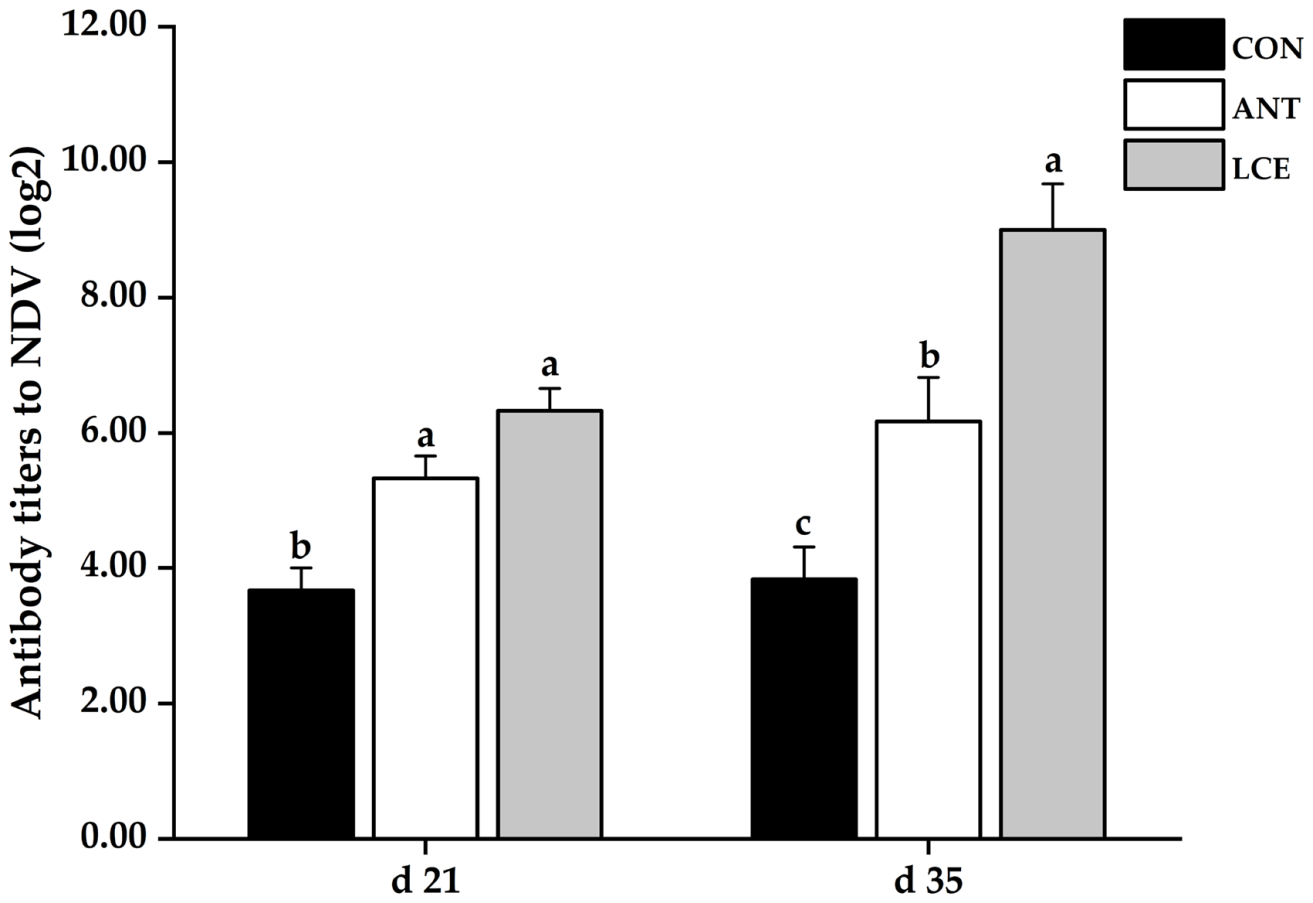
Effects of dietary LCE supplementation on serum antibody titers to NDV in broiler chickens. CON, basal diet; ANT, basal diet supplemented with 50 mg/kg spectinomycin hydrochloride and 25 mg/kg lincomycin hydrochloride; LCE, basal diet supplemented with 500 mg/kg LCE. Values are expressed as mean ± SEM, *n* = 5. ^a–c^Means without a common superscript differ significantly (*p* < 0.05).

Effects of dietary LCE supplementation on serum antibody titers to NDV in broiler chickens are shown in Figure 3. At day 21, dietary supplementation of ANT and LCE significantly increased serum antibody titers to NDV when compared with CON. At day 35, dietary treatments significantly (*p* < 0.05) affected serum antibody titers to NDV. The highest serum antibody titers to NDV were observed in birds fed LCE, followed by those fed ANT, and then those fed CON (*p* < 0.05).

### Serum inflammatory cytokines

Effects of dietary LCE supplementation on serum inflammatory cytokines in broiler chickens are presented in Table 4. At day 21, serum IL-6 concentration was reduced (*p* < 0.01) in birds fed LCE diet than those fed CON diet, but it was similar to those fed ANT diet. Both ANT and LCE supplementation decreased (*p* < 0.01) serum concentrations of TNF-α, IFN-γ and PGE-2. Supplementation of LCE increased (*p* < 0.01) serum IL-10 concentration compared with the other groups. At day 35, birds in ANT and LCE groups showed lower (*p* < 0.01) serum concentrations of IL-6 and IFN-γ than those in CON group. Supplementation of LCE increased (*p* < 0.01) IL-10 concentration and decreased (*p* < 0.01) PGE-2 concentration in serum compared with CON and ANT groups. Serum TNF-α concentration was lower (*p* < 0.01) in birds fed ANT diet than those fed CON and LCE diets.

**Table 4:** Effects of dietary LCE supplementation on serum inflammatory cytokines in broiler chickens.

Items	CON	ANT	LCE	SEM	*p*-value
*Day 21*					
IL-6 (pg/mL)	3.86^a^	3.55^ab^	3.37^b^	0.09	<0.01
IL-10 (pg/mL)	15.28^b^	14.14^c^	18.27^a^	0.08	<0.01
TNF-α (pg/mL)	18.68^a^	16.14^c^	17.81^b^	0.17	<0.01
IFN-γ (pg/mL)	11.48^a^	9.99^b^	10.19^b^	0.28	<0.01
PGE-2 (pg/mL)	225.63^a^	217.66^b^	173.26^c^	2.05	<0.01
*Day 35*					
IL-6 (pg/mL)	5.31^a^	4.65^b^	4.15^c^	0.05	<0.01
IL-10 (pg/mL)	15.23^b^	15.51^b^	17.14^a^	0.13	<0.01
TNF-α (pg/mL)	17.94^a^	16.06^b^	17.87^a^	0.13	<0.01
IFN-γ (pg/mL)	10.06^a^	8.02^c^	8.54^b^	0.14	<0.01
PGE-2 (pg/mL)	230.57^a^	230.66^a^	171.91^b^	2.07	<0.01

^a–c^Means in the same row without a common superscript differ significantly (*p* < 0.05). CON, basal diet; ANT, basal diet supplemented with 50 mg/kg spectinomycin hydrochloride and 25 mg/kg lincomycin hydrochloride; LCE, basal diet supplemented with 500 mg/kg LCE.

### Liver antioxidant capacity

**Figure 4:**
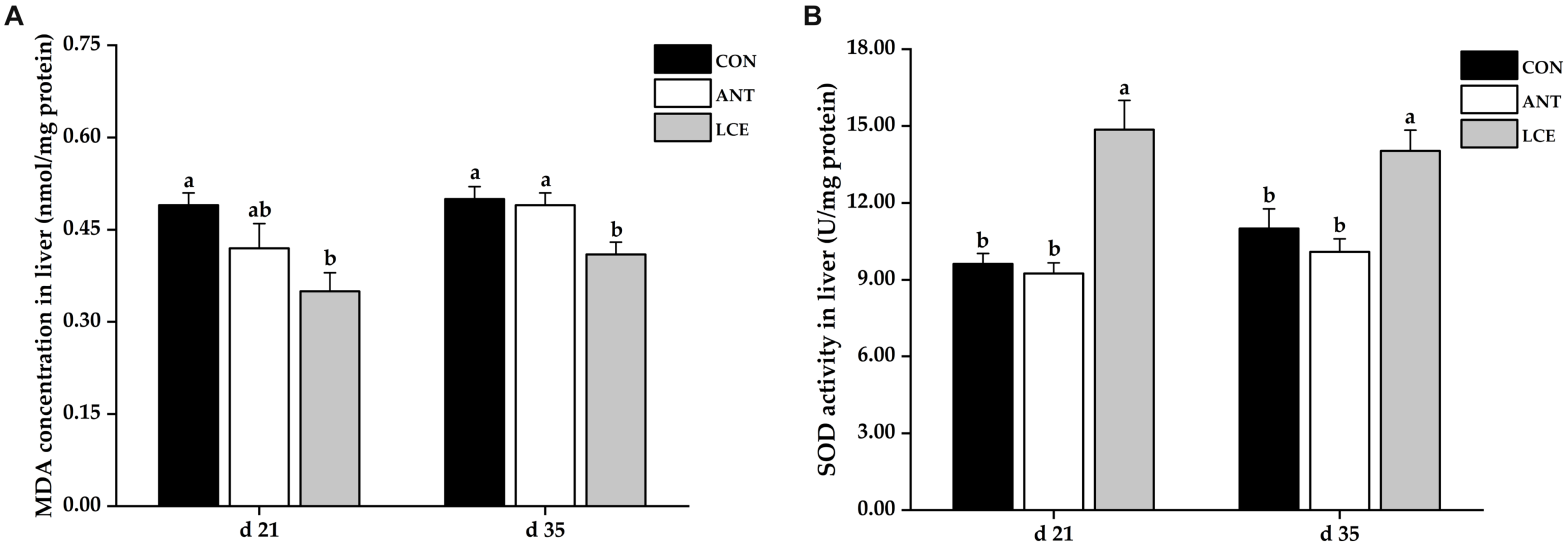
Effects of dietary LCE supplementation on liver antioxidant capacity in broiler chickens. CON, basal diet; ANT, basal diet supplemented with 50 mg/kg spectinomycin hydrochloride and 25 mg/kg lincomycin hydrochloride; LCE, basal diet supplemented with 500 mg/kg LCE. **(A)** MDA concentration in liver; **(B)** SOD activity in liver. Values are expressed as mean ± SEM, *n* = 5. ^a,b^Means without a common superscript differ significantly (*p* < 0.05).

The results of liver antioxidant capacity in broiler chickens are shown in Figure 4. Birds in LCE group had lower (*p* < 0.05) MDA concentration in liver than those in CON group at day 21, and than those in CON and ANT groups at day 35 (Figure 4A). Dietary LCE supplementation significantly increased (*p* < 0.05) liver SOD activity compared with CON and ANT groups at day 21 and 35 (Figure 4B).

### Liver inflammatory cytokines

The results of Inflammatory cytokine concentrations in liver are presented in Table 5. At day 21, liver IL-6 concentration was the lowest in birds fed LCE diet, followed by those fed ANT diet, and then those fed CON diet (*p* < 0.01). Liver IL-10 concentration was the highest for LCE group, but the least for CON group with intermediate values for ANT group (*p* < 0.01). Liver TNF-α concentration in birds fed LCE diet was lower (*p* < 0.01) than those fed either CON diet or ANT diet, which did not differ from each other. At day 35, supplementation of LCE and ANT reduced (*p* < 0.01) liver concentrations of IL-6 and TNF-α compared with CON group. On the other hand, birds in LCE group had higher (*p* < 0.01) liver concentration of IL-10 than those in CON and LCE groups.

**Table 5:** Effects of dietary LCE supplementation on inflammatory cytokine concentrations in liver of broiler chickens.

Items	CON	ANT	LCE	SEM	*p*-value
*Day 21*					
IL-6 (pg/g protein)	4.86^a^	4.63^b^	4.28^c^	0.04	<0.01
IL-10 (pg/g protein)	12.87^c^	14.66^b^	18.67^a^	0.25	<0.01
TNF-α (pg/g protein)	20.66^a^	20.28^a^	18.19^b^	0.26	<0.01
*Day 35*					
IL-6 (pg/g protein)	5.74^a^	5.42^b^	4.73^c^	0.05	<0.01
IL-10 (pg/g protein)	15.50^c^	16.20^b^	18.22^a^	0.15	<0.01
TNF-α (pg/g protein)	22.05^a^	20.15^b^	17.53^c^	0.18	<0.01

^a–c^Means in the same row without a common superscript differ significantly (*p* < 0.05). CON, basal diet; ANT, basal diet supplemented with 50 mg/kg spectinomycin hydrochloride and 25 mg/kg lincomycin hydrochloride; LCE, basal diet supplemented with 500 mg/kg LCE.

## Discussion

Plant-derived natural products have recently received increasing attention due to their potential role as alternatives to antibiotic growth promoters in animal production (28). Both CGA and curcumin are regarded as excellent non-toxic feed additives that enhance performance, immunity and antioxidant capacity in poultry (29, 30). In the present study, dietary ANT and LCE supplementation improved ADG and FCR during the entire experimental period, thereby contributing to greater BW at the end of the experiment. However, dietary treatments had no significant effects on ADFI, therefore the increased ADG by LCE supplementation may be due to the improved nutrient utilization. Furthermore, these performance parameters did not differ significantly between ANT and LCE groups, suggesting LCE has potential to replace antibiotics as growth promoter. Previous studies primarily focused on the effects of dietary supplementation with CGA and curcumin alone on growth performance in broilers, and scarce data are available concerning the effects of their combination. Partially similar to our results, Liu et al. (31) reported that dietary supplementation of 500 mg/kg CGA increased ADG and reduced FCR without affecting ADFI in broilers at an early age. Rajput et al. (32) also demonstrated that dietary supplementation of curcumin at 200 mg/kg improved BW and FCR during 0–42 days, but there was no significant difference on feed intake. The positive effects of curcumin on growth performance in that study were attributed to increased villus absorptive area of small intestine and the consequent improved nutrient digestibility, which may be a possible explanation for the present results.

Oxidative stress is a state of imbalance between oxidants and antioxidants, which results in damaging effects on growth and health in poultry (33, 34). The enzymatic antioxidant system, including GSH-Px, CAT and SOD, plays a crucial role in protecting organisms against oxidative stress (35). CGA and Curcumin are phenolic compounds which have been proved to have potent antioxidant activity *in vitro* and *in vivo* (21, 36, 37). In the current study, increased serum activities of GSH-Px and SOD in LCE group was observed, indicating LCE improved antioxidant capacity by enhancing antioxidant enzyme activities. MDA is a product of lipid peroxidation and widely used as an indicator of oxidative stress (38). T-AOC considers the cumulative effect of all antioxidants present in blood or body fluids (39). The results of lower serum MDA concentration and higher T-AOC in LCE group also confirmed that LCE improved serum antioxidant capacity. As the principal organ involved in various metabolic functions, the liver is prone to oxidative stress-related damages because high levels of reactive oxygen species are generated during metabolic processes (40). In this study, lower MDA concentration and higher SOD activity in liver by LCE supplementation suggested a better antioxidant status, which were consistent with the results obtained in serum samples. Our results were similar to previous studies showing that dietary supplementation of CGA or curcumin increased SOD and GSH-Px activities and reduced MDA concentration in serum of broilers under high stocking density stress (14, 41). Taken together, these observations demonstrate that dietary LCE supplementation can enhance antioxidant function in broilers.

Immunity is categorised to innate (non-specific) and adaptive (specific), which work closely together to defense against pathogens (42). Innate immunity is not only the first line of defense against various invading pathogens, but also the stimulus for the adaptive immunity (43). Adaptive immunity is the antigen-specific immune responses mediated by B and T lymphocytes, including the production of antibodies (44). In the present study, both innate and adaptive immunity were evaluated by investigating the phagocytic activity and antibody titers to NDV, respectively. The results showed that the phagocytic index and serum antibody titers to NDV in birds fed LCE diet were higher than those fed CON diets, suggesting dietary supplantation of LCE could improve innate and adaptive immunity in broiler chickens. He et al. (45) demonstrated that CGA notably enhanced macrophages phagocytosis both *in vitro* and *in vivo*. Rajput et al. (46) also found that dietary supplementation with curcumin enhanced antibody response as evidenced by increased serum antibody titers to ND in broiler chickens. Therefore, the improved immunity observed in this study may be attributed to the immunomodulatory activity of CGA and curcumin in LCE.

Cytokines are pleiotropic polypeptides released by immune cells involved in various biological processes (47). Pro-inflammatory cytokines such as IL-6, TNF-α, and IFN-γ are involved in the up-regulation of inflammatory reactions, while anti-inflammatory cytokines such as IL-10 prevent over exuberant inflammation (48). In the present study, lower serum concentrations of pro-inflammatory cytokines including IL-6, TNF-α and IFN-γ and higher serum concentrations of pro-inflammatory cytokine IL-10 were observed in birds fed LCE diet when compared with those fed CON diet. The same trends were also observed in liver, suggesting less systemic inflammation. Similarly, anti-inflammatory effects of CGA or curcumin has also been reported in previous studies with broilers (41, 49). As an anti-inflammatory cytokine, IL-10 has potent anti-inflammatory properties, inhibiting the expression of pro-inflammatory cytokines such as IL-6 and TNF-α at multiple levels (48). Therefore, the decreased production of pro-inflammatory cytokines may be associated with the increased concentration of IL-10. PGE-2 is recognized as a potent proinflammatory mediator and plays a crucial role in various inflammatory diseases (50). The current results also revealed that LCE decreased serum concentration of PGE-2, again confirming reduced inflammatory response.

Taken together, these results indicated dietary supplementation of 50 μg/kg chlorogenic acid and 10 mg/kg curcumin is safe and effective in terms of the improved growth and immunity of broilers. At present, researches mainly focus on dosage effects of CGA or curcumin alone (14, 20–22). The recent study has shown that CGA and curcumin are synergic in biologic activities, suggesting the concentrations of CGA and curcumin can be reduced when they are used in combination. Therefore, dosage effects of CGA and curcumin in combination should be evaluated to determine optimal dose ranges in further study.

## Conclusion

In conclusion, dietary supplementation of LCE improved growth performance by enhancing antioxidant capacity, strengthening immune system and alleviating inflammation, which has potential as antibiotic alternatives.

## Data availability statement

The original contributions presented in the study are included in the article/supplementary material, further inquiries can be directed to the corresponding authors.

## Ethics statement

The animal study was approved by Institutional Animal Care and Use Committee of Hebei Agricultural University. The study was conducted in accordance with the local legislation and institutional requirements.

## Author contributions

DX: Data curation, Formal analysis, Investigation, Methodology, Resources, Writing – original draft. XW: Data curation, Formal analysis, Investigation, Validation, Writing – original draft. WS: Conceptualization, Supervision, Writing – review & editing. YB: Conceptualization, Supervision, Writing – review & editing.
